# Genetic association between 1425G/A SNP in PRKCH and hypertrophic cardiomyopathy in a Chinese population

**DOI:** 10.18632/oncotarget.22214

**Published:** 2017-10-31

**Authors:** Feng Ji, Qun Liu, Zeyu Feng, Xinwei Han, Zhitong Li

**Affiliations:** ^1^ Department of Interventional Radiology, The First Affiliated Hospital of Zhengzhou University, Zhengzhou 450052, China; ^2^ Johns Hopkins University School of Medicine, Baltimore, MD 21224, USA; ^3^ Medical School of Nantong University, Nantong 226001, China

**Keywords:** hypertrophic cardiomyopathy, genetics, polymorphism, hypertrophic obstructive cardiomyopathy, PRKCH

## Abstract

Hypertrophic cardiomyopathy is a heterogeneous myocardial disorder with a broad spectrum of clinical presentation and morphologic features. Previous reports indicated that protein kinase C pathway as a major determinant of cardiac hypertrophy and heart failure. Population-based analyses of the association between PRKCH gene (encoded PKCη) and HCM has not been performed yet. The purpose of this study is to investigate the association of the nonsynonymous SNP (1425G/A) in PRKCH gene and hypertrophic cardiomyopathy in a Chinese population. 323 patients with HCM and 326 controls were examined using a case-control methodology. The 1425G/A SNP in PRKCH was genotyped by allele-specific real-time PCR assay. The 1425G/A SNP in PRKCH increased the risk of HOCM (hypertrophic obstructive cardiomyopathy) (OR=1.427, 95% confidence interval, 1.013 to 2.012, P=0.046) under a dominant model. After age- and sex-adjustment, the significant associations remained in HOCM (for GG +AG versus AA, OR= 2.497, 95% confidence interval, 1.01 to 6.17; P=0.047). The 1425G/A SNP in PRKCH increases the risk of hypertrophic obstructive cardiomyopathy in the Chinese population.

## INTRODUCTION

Hypertrophic cardiomyopathy (HCM) is the most commonly primary autosomal dominant inherited myocardial disease characterized by unexplained myocardial hypertrophy leading to progressive ventricular dysfunction and sudden cardiac death (SCD) [1]. As a global disease, HCM may affect groups of all age ethnicity, with an estimated prevalence more than 1 in 500 people worldwide [2]. Recognition of HCM is important, both for providing treatment and prevention strategies and in triggering the initiation of clinical and genetic surveillance of family members [3].

To date, over 1,400 responsible mutations have been documented in more than 25 genes [4], the most important genes encode the protein components of the cardiac sarcomere, which perform contractile, structural and regulatory functions. These include thick filament proteins (*MYH7*, *MYL2*, and *MYL3*), thin filament proteins (*TNNT2*, *TNNI3*, *TNNC1*, *TPM1*, and *ACTC*), intermediate filament proteins (*MYBPC3*), and Z-disc proteins (*ACTN2*, *MYOZ2*). Mutations in the myosin heavy chain (*MYH7*) and myosin-binding protein C (*MYBPC3*) are the most common and account for roughly 80% of sarcomeric HCM [5]. With increased understanding of the molecular genetic causes of HCM and advances in modern laboratory technology, clinical genetic testing for HCM has become increasingly feasible [1]. However, HCM is clinically heterogeneous, with inter- and intra- familial variations ranging from benign forms to malignant forms with a high risk of cardiac failure or SCD [5], biochemical and biophysical analyses have shown that there is no unifying abnormality of cardiac contractility resulting from mutations of identified causal genes [6, 7]. Patients carrying the same mutation have a considerably different clinical spectrum and prognosis in the inter- and intra-family [8–10]. This suggest that the phenotype of HCM is not simply a direct consequence of altered contractility and a more fundamental abnormality of myocardial function, new variants and environmental factors should be explored.

In genome wide association studies, the PRKCH gene, which encodes PKCƞ, has been reported as a novel susceptibility gene for atherosclerotic diseases such as cerebral infarction [11] and was involved in the development and progression of atherosclerosis in humans [12]. Observations in cell culture and animal models implicated protein kinase C (PKC) as an important signal transduction pathway in the development of cardiac hypertrophy [13]. PKC is a serine-threonine kinase that regulates a wide variety of important cellular functions including proliferation, differentiation and apoptosis [13]. A 1425G/A SNP (single nucleotide polymorphism) (leading to V374I) of PRKCH gene which lies in exon 9 and within the ATP-binding site of PKCη enhances the kinase activity [14]. Therefore, we focused on the association of the nonsynonymous SNP (1425G/A) in PRKCH gene and HCM in an independent case-control sample.

## RESULTS

Between February 2011 and October 2013, 323 HCM cases and 326 controls were enrolled. Of the cases, 60.7% were male, with mean age of 55.9±16.7 years. The characteristics of the study participants are shown in Table 1. The ratio of age, sex, weight, height and blood pressure, we calculated the BMI (body mass index) of individuals at the first interview (baseline) and prevalence of diabetes in cases were not different from those in controls. DBP (diastolic blood pressure) were lower in cases than those of in controls.

**Table 1:** Basic Characteristics of Study Participants

	HCM Patients (n=323)	Controls (n=326)	P Value
Age (years)	55.87±16.65	56.22±14.71	0.758
Male (%)	60.70	63.20	0.282
BMI (kg/m^2^)	24.93±3.15	25.04±3.17	0.699
SBP (mmHg)	123.82±20.46	124.20±13.58	0.806
DBP (mmHg)	74.93±12.12	79.89±9.75	0.000
DM (%)	5.9	7.4	0.275

^*^ BMI = weight (Kg)/height (m)^2^; SBP: systolic blood pressure; DBP: diastolic blood pressure.

The percentage of patients with hypertrophic obstructive cardiomyopathy (HOCM) and non-obstructive cardiomyopathy is 70.6% (228) and 29.4% (95) of overall patients respectively. We genotyped the 1425G/A SNP in PRKCH (rs2230500) in the cohort (Table 2). The SNP was in Hardy–Weinberg equilibrium (P> 0.05). Associations between the 1425G/A SNP in PRKCH and different types of HCM are shown in Table 2. The allele of A increased the risk of HOCM (OR=1.43, P=0.046) whereas no significant association can be found between this allele and non-obstructive cardiomyopathy (OR=1.108, P=0.781).

**Table 2:** Case-Control Study Showing Association between the 1425G/a SNP in PRKCH and HCM

Samples	Case					Control					MAF		Unadjusted (A/G)	
	AA	AG	GG	Sum	H-W	AA	AG	GG	Sum	H-W	Case	Control	Odds Ratio 95% CI	P Value
Screening														
HCM	16	95	206	317	0.250	7	86	233	326	0.775	0.200	0.153	1.383 (1.036, 1.845)	0.028
Obstructive	11	69	144	224	0.468	4	62	165	231	0.505	0.203	0.152	1.427 (1.013, 2.012)	0.046
Non-obstructive	5	22	66	93	0.102	3	24	68	95	0.626	0.172	0.158	1.108 (0.691, 1.718)	0.781

For further clarify the independent effect of the 1425G/A SNP in PRKCH, we performed multivariate analysis with adjustment for demographic factors using a conditional logistic regression model in 228 individuals with HOCM along with age- and sex-matched controls. Odds ratios for the incidence of HOCM were shown in Table 3. The 1425G/A SNP in PRKCH increased the risk of HOCM (age- and sex-adjusted odds ratio= 3.41; 95% CI, 1.05 to 11.05; P=0.041) under a dominant model. For 1425G→A, age- and sex-adjusted odds ratio of AA genotype was 2.49 (95% CI, 1.01 to 6.17; P=0.047) under a recessive model.

**Table 3:** Odds Ratios for the Incidence of Hypertrophic Obstructive Cardiomyopathy

Samples	Genotype of the 1425G/A SNP	No. of Case	Total No. of Subjects	Age- and Sex-Adjusted	
				Odds Ratio (95% CI)	P Value
	GG	144	309	1.00	
	AG	69	131	1.32 (0.87, 2.00)	0.196
	AA	11	15	3.41 (1.05, 11.05)	0.041
	GG+AG	213	440	1.00	
	AA	11	15	2.50 (1.01, 6.17)	0.047

## DISCUSSION

In the present study, we examined the 1425G/A SNP in PRKCH with HCM in the Chinese population. This SNP was previously shown to increase the risk of ischemic stroke and cerebral hemorrhage in Chinese [11] and Japanese population [15], and a higher risk genotype for severe gastric atrophy [16]. Recent research implicates PKC activation in the pathophysiology of a number of cardiovascular disease states [13, 17]. Our results showed the first time that the 1425G/A SNP in PRKCH increases the risk of HOCM (Table 2 and Table 3) and this association remains significant under dominant and recessive models after age- and sex-adjustment.

The gene PRKCH encodes protein kinase C, which is a family of serine- and threonine-specific protein kinases that can be activated by calcium and the second messenger diacylglycerol. PKC family members phosphorylate a wide variety of protein targets and are known to be involved in diverse cellular signaling pathways and regulates multiple important cellular functions, including proliferation, differentiation, and apoptosis [13]. Research data from conventional animal models, genetically engineered mice and human myocardium implicate activation of the PKC pathway as a major determinant of cardiac hypertrophy and heart failure [18]. Even though there are species-specific differences present in the differential PKC isoform response, the action of PKC in general is a common pathway in the development of cardiac hypertrophy [18].

PRKCH is located in chromosome 14q22-q23 in human and the 1425G/A SNP (leading to Val374Ile) is located within an ATP-binding site (exon 9) of PKCη enhances the kinase activity [19]. Previous studies showed that the nonsynonymous SNP in PRKCH increases the risk of stroke in the Japanese and Chinese population [15, 19] and confer increased risk of RA through aberrant T cell-mediated autoimmune responses [20], and act as a higher risk genotype for severe gastric atrophy due to the dysregulation of PKCη signal transduction pathway(s) [16]. Contrary to other PKCs, which are primarily enriched in the brain tissue, PKCη is mainly expressed in lung, skin and heart tissues [21]. PKCη participates in various cellular processes including proliferation, differentiation, secretion and apoptosis [22–24]. Recent reports have revealed the role of PKCη in immune function [25]. PKCη was shown to be important for T-cell proliferation and homeostasis [26], and was also implicated in the regulation of toll-like receptor-2 (TLR-2) responses in macrophages [27]. Centurione et al reported PKCη regulated the hypertrophic and apoptotic events in rat neonatal heart through regulating NF-kB signaling system and intrinsic mitochondrial apoptotic route of rat life [28].

Noteworthy, results from our study showed the 1425G/A SNP in PRKCH increased the risk of HOCM under a dominant model whereas no statistical significance can be observed between 1425G/A SNP in PRKCH and non-HOCM. These findings are in agreement with the well-recognized knowledge that left-ventricular outflow tract obstruction (LVOTO) is a hallmark of a worse prognosis [29]. Furthermore, our study indicate the 1425G/A SNP in PRKCH as a biomarker for definition of differences in presenting features and long-term mortality between non-HCM and HOCM.

However, some limitations should be kept in mind in this work. First, the number of subjects is relatively small in non-HOCM treatment group, and it may have insufficient statistical power to determine the relationship between PRKCH 1425G/A and non-HOCM. In addition, the mechanism and the signaling pathway of PRKCH are very complex. Thus, more molecular and cellular experiments should be performed to further illuminate the mechanism involved.

In conclusion, our study showed that the 1425G/A SNP in PRKCH were significantly associated with HOCM in a Chinese population suggesting the PRKCH gene encoding PKCη as a putative candidate gene conferring genetic susceptibility to HOCM in a Chinese population. Although PRKCH certainly has biologic plausibility as an HOCM gene, replication studies in other independent populations and/or by family-based tests of association are essential to confirm the observations. Further investigations into the molecular mechanisms by which PRKCH alters HOCM susceptibility are also required.

## MATERIALS AND METHODS

### Subjects recruitment

The HCM patients of this study were recruited from the First Affiliated Hospital of Zhengzhou University during 2010 to 2013. All patients had hypertrophic cardiomyopathy as diagnosed by echocardiograms showing unexplained left ventricular hypertrophy. Inclusion criteria included ≥13 mm thickness of the left ventricular wall and ≥1:3 of the ratio of interventricular septum thickness to posterior wall thickness of the left ventricle with unknown causes of LVH shown by echocardiography [1]. Left ventricular outflow obstruction was defined as a peak instantaneous gradient at least 30 mmHg under basal (resting) conditions attributable to systolic anterior motion (SAM) of the mitral valve. Exclusion criteria included secondary cardiac hypertrophy related to other cardiovascular diseases, such as hypertension, valvar heart disease, pulmonary hypertension, coronary heart disease and metabolic diseases that may cause LVH revealed by medical history and physical examination, echocardiography or coronary angiography. Of the 323 patients, 262 had sporadic HCM and 61 had family history of HCM.

Control subjects were selected according to the case control study criteria in the same geographic location (control subjects matched to cases by sex, age and blood pressure), who had no history or symptoms of cardiovascular diseases shown by medical history, physical examination and echocardiography.

The study was approved by Institutional Review Borad of the First Affiliated Hospital of Zhengzhou University and informed consent were obtained from all study participants upon enrolment.

### Genotyping

Totally, 649 participants (323HCM cases and 326 controls) were recruited and blood samples were collected after a 12-hour overnight fast and stored at −70°C until use. Biochemical variables including serum sodium, potassium, creatinine, uric acid, blood urea nitrogen (BUN), total plasma cholesterol, triglyceride, high density lipoprotein cholesterol (HDL-C), low density lipoprotein cholesterol (LDL-C), and blood glucose were analyzed according to standard protocols.

Genomic DNA was extracted from cellular buffy coat using QIAamp DNA Blood Midi Kit. The 1425G/A SNP in PRKCH (rs2230500) was genotyped by allele-specific real-time polymerase chain reaction using Gene-Amp 5700 Sequence Detector (Applied Biosystem).

### The PCR amplifications were performed using the following primers

Common primer, 5′-GCAGAATCACGTCCTTC TTCAG-3′-;

Allele-specific primer (A), 5′-CATAGGTGATGC TTGCAAGAA-3′;

Allele-specific primer (G), 5′-CATAGGTGATGC TTGCAAGAG-3′;

Individual DNA sample was genotyped for single SNP by using an equal aliquot of samples with 2 allele-specific PCR reactions, each containing 1 of the allele-specific (A-S) primers and a common primer. PCR reaction with the A-S primer that matched the allele in the template DNA amplified normally, whereas PCR reaction with the other A-S primer that mismatched the allele in the template was prevented or delayed when PCR reaction was monitored in real-time (by including SYBR Green I in the PCR and following fluorescence cycle-by-cycle). For each amplification, a fluorescence threshold near the baseline fluorescence was used to calculate a cycle threshold value, which was then used to call the genotype of the sample. PCR was carried out on the GeneAmp 5700 Sequence Detector with procedure of 12 minutes at 95°C, followed by 45 cycles of 30 seconds at 95°C, 30 seconds at 58°C, and finished by 20 minutes dissociation at 60°C. Genotype was directly obtained with the GeneAmp 5700 SDS software. The genotyping call rate was 98% and the concordance between our genotyping and direct sequencing was 100% by direct sequencing 100 random samples.

### Statistical analysis

Clinical data about continuous variables expressed as mean±SD, and differences between groups were assessed by Student’s t-test. Categorical variables were represented as percentage and were tested by X^2^ analysis. Hardy–Weinberg equilibrium was also assessed by X^2^ analysis. Our analyses concerned the whole study group and were subsequently stratified by 2 major types of HCM, HOCM and non-HOCM. In each stratum, cases were compared with the corresponding control groups. Odds ratios in dominant model with corresponding 95% confidence intervals were computed by the Woolf’s method to test the effect of genotype on HCM risk. Adjusted odds ratios for age and sex were performed by multiple logistic regression with genotypes, age and sex as the independent variables. Data were analyzed using SAS statistical software (version 9.1, SAS Institute Inc). P< 0.05 was used to indicate statistically significant differences.

## Abbreviations

HCM: hypertrophic cardiomyopathy
SCD: sudden cardiac death
PKC: protein kinase C
HOCM: hypertrophic obstructive cardiomyopathy
LVOTO: left-ventricular outflow tract obstruction
